# Functional insight into LOAD-associated microglial response genes

**DOI:** 10.1098/rsob.210280

**Published:** 2022-01-26

**Authors:** Lauren A. Jonas, Tanya Jain, Yue-Ming Li

**Affiliations:** ^1^ Weill Cornell, Weill Graduate School of Medical Sciences of Cornell University, New York, NY 10065, USA; ^2^ Chemical Biology Program, Memorial Sloan Kettering Cancer Center, New York, NY 10065, USA

**Keywords:** microglia, LOAD, Alzheimer's, TREM2

## Abstract

Alzheimer's disease (AD) is characterized by the presence of amyloid beta (Aβ) plaques and neurofibrillary tangles (NFTs), neuronal and synaptic loss and inflammation of the central nervous system (CNS). The majority of AD research has been dedicated to the understanding of two major AD hallmarks (i.e. Aβ and NFTs); however, recent genome-wide association studies (GWAS) data indicate neuroinflammation as having a critical role in late-onset AD (LOAD) development, thus unveiling a novel avenue for AD therapeutics. Recent evidence has provided much support to the innate immune system's involvement with AD progression; however, much remains to be uncovered regarding the role of glial cells, specifically microglia, in AD. Moreover, numerous variants in immune and/or microglia-related genes have been identified in whole-genome sequencing and GWAS analyses, including such genes as *TREM2*, *CD33*, *APOE*, *API1*, *MS4A*, *ABCA7*, *BIN1*, *CLU*, *CR1*, *INPP5D*, *PICALM* and *PLCG2*. In this review, we aim to provide an insight into the function of the major LOAD-associated microglia response genes.

## Graphical abstract

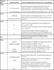

## 1. Background

### 1.1. Late-onset Alzheimer's disease and immune risk

Alzheimer's disease (AD) is the most common neurodegenerative disorder and the most prevalent cause of dementia. It is currently estimated to affect more than 5 million people and is the sixth leading cause of death in the United States [1]. Likely beginning decades before symptoms of cognitive impairment first manifest, AD pathology is classified by the accumulation of extracellular amyloid beta (Aβ) plaques and intracellular hyper-phosphorylated tau tangles. Underlying these hallmarks are glial cell activation and neuroinflammation, synaptic dysfunction and ultimately neurodegeneration and brain atrophy [2]. Until recently, neuroinflammation and innate immune activation were assumed to play a purely responsive role to AD pathology; however, recent genomic data have provided a framework of support for the causative role of immune cells in AD development.

Generally, AD can be classified into two groups based on the age of onset. Less than 1–2% of AD cases are of a familial nature and can present as early onset before the age of 65 with a rapid onset of disease progression [3,4]. Identification of these familial mutations in amyloid precursor protein (APP) and presenilin-1 (PSEN1, the catalytic subunit of γ-secretase involved in APP cleavage) has provided immense insight into disease aetiology [5]. Conversely, the majority of AD cases are classified as sporadic or late-onset AD (LOAD), affecting individuals greater than 65 years of age. Age is the biggest risk factor for developing LOAD [6,7]. In addition, the presence of recently identified LOAD-risk alleles has shown to play a significant role in AD development, with a heritability estimate of 60–80% [8].

**Figure 1.**
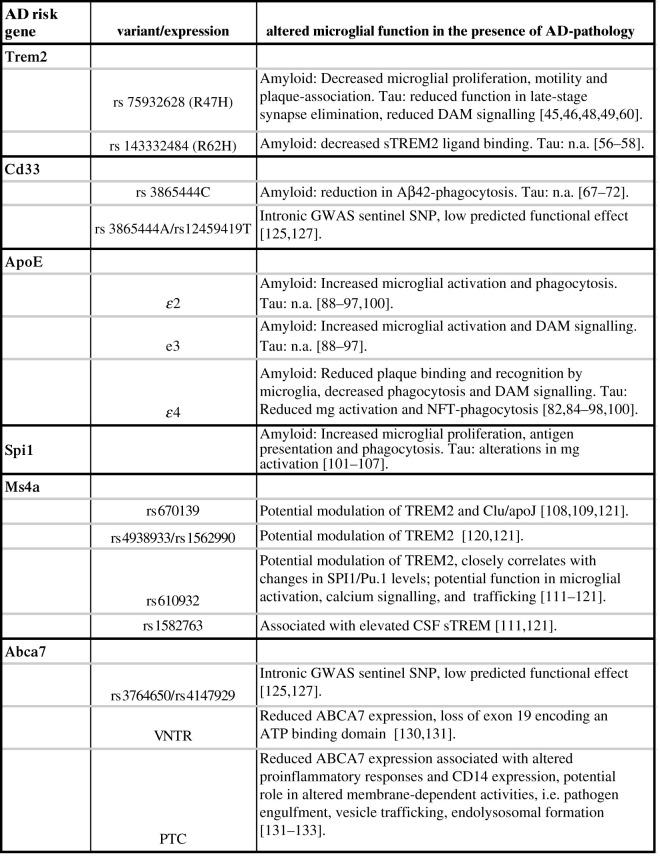
AD risk variants and associated effect on altered microglial function in the presence of AD pathology. CSF, cerebrospinal fluid; DAM, disease-associated microglia; SNP, single nucleotide polymorphism.

Genome-wide association studies (GWAS) and genetic linkage studies over the past decade have helped identify numerous allelic loci associated with LOAD. The alleles identified in these studies are more common in the general population but are less penetrant and confer a smaller risk of developing AD, as compared to familial AD mutations [9,10]. These studies, as well as early histological data from patient brain tissue, have provided considerable evidence for the involvement and activation of the immune system in AD pathology [11,12]. Moreover, recent whole-genome sequencing methods have highlighted many immune-related genes and variants as risk factors for AD, including *TREM2*, *CD33*, *APOE*, *API1*, *MS4A*, *ABCA7*, *BIN1*, *CLU*, *CR1*, *INPP5D*, *PICALM* and *PLCG2*. Of the more than 40 identified risk variants for AD, a majority of risk alleles are enriched in myeloid and microglia cell enhancers, underscoring significant microglial involvement in AD disease progression (figure 1) [13–19]. While identified variants confer only a small contribution to AD compared to AD risk genes *APP* and *PSEN1*, these studies emphasize the significance of microglial involvement in disease development [10,13]. Genomic and functional studies indicate that microglia not only play a reactionary role in AD pathology, but also are themselves a causative factor in initial AD development and progression.

However, while genetic data are critical for the initial identification of microglial involvement in AD, research remains necessary to unveil specific functions through which these cells confer risk for AD. Since the identification of these microglial-related LOAD-associated genes, much research has gone into providing insight into the function of these genes. Ultimately, understanding microglial-specific roles in AD will allow for the development of novel disease-modifying therapies. This review aims to provide functional insight into LOAD-associated microglial genes.

### 1.2. Microglia in AD

Microglia, the brain-resident macrophages, play critical roles in central nervous system (CNS) innate immunity. Microglia are key players in the immune response, which can be briefly summarized as (i) initial pathogen surveillance, (ii) phagocytosis and (iii) degradation and responsive signalling [20–22]. Functional studies highlight various genes as they contribute to these distinct processes within the immune response and further, changes in protein function throughout different stages of disease progression.

Recent studies have been directed at unveiling the distinct ways in which microglia are involved in AD-related immune processes. As stated previously, microglial contribution to AD can be summarized into distinct stages, the first of which is *initial pathogen surveillance*. This first step is initiated when distinct signalling molecules (i.e. pathogens, dystrophic neurites, protein aggregates) identify and bind target-recognizing receptors (i.e. TREM2, CD33) on microglia. Distinct ligand–receptor combinations drive differential signalling, resulting in the modulation of various functions [21]. For example in the case of AD, targets such as Aβ or neurofibrillary tangles (NFTs) are recognized by toll like-receptors (TLRs) resulting in a proinflammatory cytokine storm associated with the release of cytokines or effector molecules (i.e. TNF, IL-1, NO), while recognition of cell debris or dystrophic neurites by microglial TREM2 receptors is associated with a phagocytic response along with an increase in TGFβ and IL10 signalling [23]. After initial recognition, microglial *uptake of pathogenic targets* may be initiated; the plasma membrane extends and encloses around the target forming a vesicular phagosome. This nascent phagosome subsequently fuses with lysosomes forming a phagolysosome. Lastly, *digestion* occurs within the phagolysosome where the target is degraded. Following this, byproducts must be either stored or recycled by the phagocytic cell [20]. Further, microglia respond to pathogenic targets through responsive signalling, through altered cytokine production and gene expression.

Recent evidence has highlighted a subset of microglia with a disease-associated gene signature. Disease-associated microglia (DAM), were first identified through a comprehensive single-cell RNA sequencing analysis of immune cells isolated from the 5xFAD mouse model of AD [24]. These cells express microglial markers such as *Iba1*, *Cst3* and *Hexb*, but have downregulation of homeostatic markers like *P2ry12, P2ry13, Cx3cr1, CD33* and *Tmem119* [25]. Furthermore, they display an upregulation of *Trem2*, *Tyrobp*, *Ctsd*, *Apoe* and *Lpl*, which are genes involved in phagocytic and lysosomal functions of microglia, as well as lipid metabolism [9]. Subsequent studies have identified similar DAM profiles in human AD post-mortem tissue, as well as in other mouse models of neurodegeneration including APP/PS1, PS2APP, tau P301 L and P301S, ALS mouse models, MS models and ageing mouse models. Alterations in *DAM* gene expression are associated with differential microglial function [26–33].

## 2. Microglial risk factors and their functional relevance

### 2.1. TREM2

TREM2 is an immunoreceptor expressed on myeloid cells, including microglia. Whole-genome sequencing identified rs75932628, the *TREM2* R47H variant, in 2013. This is thought to be a loss-of-function single nucleotide polymorphism (SNP) and increases the risk of developing AD by approximately 2- to 4-fold [34–36]. Additionally, higher levels of soluble TREM2 (sTREM2) in the cerebrospinal fluid (CSF) of patients with AD carrying this SNP has been correlated with disease progression [37]. Rs143332484 (R62H) was identified in 2014 as a significant risk modifier for AD [38], but its function in AD progression is not known. There is some evidence that sTREM2 containing either SNP that causes an arginine to histidine substitution is less effective than WT sTREM2 at activating microglia and promoting survival [39]. Both SNPs are known to result in reduced stability and impaired ligand binding. Additionally, two novel transcripts apart from the canonical long-form of *TREM2* have been identified in human post-mortem brains that may add to the functional relevance of *TREM2* in AD [40].

Though the physiological function of TREM2 is not completely understood, reported TREM2 ligands include lipidated apolipoprotein E (APOE), an aforementioned AD-associated gene and Aβ oligomers, both of which are components of amyloid plaques [14,41–43]. TREM2 signalling pathways, specifically via *TYROBP*/DAP12, underscore its role in a number of microglial-related cellular functions including inhibition of proinflammatory signalling and phagocytic uptake, as well as cell proliferation and survival [44]. High-resolution microscopy has revealed that TREM2 and DAP12 are highly concentrated in processes adjacent to Aβ plaques in the AD brain, suggesting an enrichment of ‘active’ TREM2 signalling [14,45]. DAP12 is an adaptor protein that associates with TREM2, and contains an immunoreceptor tyrosine-based activation motif (ITAM) [46]. Upon ligand binding, the ITAM is phosphorylated, leading to the recruitment of spleen tyrosine kinase (Syk). Subsequent downstream signalling via activation of phosphatidylinositol 3-kinase (PI3 K) and mitogen-activated protein kinases (MAPKs) has been shown to result in elevation of intracellular Ca^2+^ through the release of IP3-gated Ca^2+^ stores and subsequent signalling cascade [47].

Initially, the AD-associated *TREM2* mutations were assumed to result in a loss-of-function phenotype. *In vitro* experiments have shown that TREM2^−/−^ microglia have impaired phagocytic capacity [48]. Several *in vivo* studies have shown that loss of TREM2 function or the presence of the R47H allele in amyloid-dependent AD mouse models increases plaque seeding and restricts the ability of microglia to proliferate and physically associate with plaques to form a microglia barrier [49]. This can lead to a reduction of plaque compaction leading to a diffuse plaque morphology, and a subsequent increase in neuritic dystrophy [45,47,50,51]. Interestingly, other studies using younger mice have shown that a TREM2 deficiency early on reduces Aβ pathology [51,52]. Further, a lack of TREM2 seems to worsen the phenotype of an amyloid-dependent AD mouse model but not their WT littermates, and this outcome is also based on their APOE genotype [41]. Conversely, elevated TREM2 expression in the 5xFAD mouse model has been shown to reduce the amyloid burden and improve memory performance. TREM2-overexpressing microglia also show a dampening of proinflammatory gene expression and an upregulation of many genes linked to phagocytosis, and are also seen to be more phagocytic *in vitro* [32,53]. TREM2-mediated phagocytosis is critical for Aβ and neuronal debris clearance in AD [43,48]. These findings consistently suggest that TREM2 signalling is important for microglial phagocytosis, proliferation and recognition and clustering around plaques, but the exact mechanism through which TREM2 affects AD pathology may be disease stage-dependent [32,37,42–44,53,54].

In addition to full-length *TREM2*, there is increasing evidence that sTREM2 may also be involved in microglial dynamics and response to AD pathology. sTREM2 is the soluble form of TREM2 produced by proteolytic cleavage of TREM2 by metalloproteases ADAM10/17 [37,48]. *In vitro* data suggest sTREM2 enhances microglial viability and helps trigger inflammatory responses by activating the Akt–GSK3β–β-catenin and NF-κB signalling pathways [54]. Levels of sTREM2 have been found to be elevated in AD, and carriers of the R47H *TREM2* mutation present even higher levels of CSF sTREM2 while maintaining similar surface expression on cells. Intriguingly, a recent study found a correlation between higher CSF sTREM2 levels and an attenuation of risk of future cognitive decline in APOE4 carriers [55]. This suggests that sTREM2 may be protective and that signalling via sTREM2 may be yet another pathway through which TREM2 can influence neuroinflammation and AD pathology [37,56,57].

Taken together, this body of evidence indicates that TREM2 signalling via DAP12 is necessary for the recognition of toxic species like Aβ, initiating microglial activation in the AD brain, as well as enhancing proliferation and survival of microglia leading to a sustained microgliosis response. This, in turn, is crucial for phagocytosis of Aβ and clearance of neuronal debris. This also brings to light the fact that certain features of immune activation are indeed beneficial in the context of neurodegenerative disease, and that the response itself depends on the timeline of disease progression.

In parallel, TREM2's effect on tau-related disease progression has also been investigated. Importantly, levels of sTREM2 in the CSF of AD patients positively correlated with total tau and *p*-tau in the CSF [37,58]. Homozygous deletion of TREM2 in PS19 mice that overexpress the human P301S mutation has been shown to be protective against neurodegeneration, as well as prevent microglia activation, without affecting tau pathology [28,59]. On the other hand, Sayed *et al.* [59] showed that TREM2 haploinsufficiency in the PS19 model confers increased brain atrophy, exaggerated tau pathology, and an increase in proinflammatory markers suggesting a dose-dependent response of TREM2 in PS19 mice. Further, TREM2^−/−^hTau mice (a less aggressive tauopathy model) were found to display decreased microgliosis, similar to TREM2^−/−^;PS19 mice; however, tau pathology was worsened. These data suggest that TREM2's contribution to pathology is disease stage dependent, and that TREM2 may play multiple roles in initial disease development versus late-stage disease progression. During the initial stages of tau pathology, in the absence of neurodegeneration (i.e. hTau mice), reduced TREM2 function is suggested to promote tau pathology, whereas decreased TREM2 function in late-stage disease progression (i.e. PS19 mice) proves to be protective against neurodegeneration. Further, mice expressing the R47H mutations exhibit reduced tau pathology and neurodegeneration [60]. Studies indicate that this effect is in large part due to alterations in microglial activation, as mice showed reduced expression of DAM genes as well as phagolysosomal marker CD68. Impaired microglial phagocytosis was hypothesized to be responsible for reduced neurodegeneration, as studies have shown that microglial TREM2 is required for synapse elimination in brain development [61]; R47H microglia showed reduced engulfment of postsynaptic elements, namely the complement protein C1q [60]. Moreover, Dejanovic *et al.* has previously reported a large increase in complement C1q in neuronal synapses of PS19 mice and AD patients, and a rescue of tau-induced synaptic loss by C1q antibodies. Synaptic C1q was found to be associated with dysregulated microglial phagocytosis of synapses *in vivo* and decreased synapse density *in vitro* [31].

### 2.2. CD33

CD33 is another transmembrane immunoreceptor expressed on myeloid cells including microglia, and another top-ranked AD-associated risk gene. *CD33* expression is found to be elevated in AD patients' brains, in microglial cells and in infiltrating macrophages [62,63]. It was first implicated in AD in 2008, when the minor allele (G) of rs3826656 was reported as a risk factor for LOAD [64,65]. The major allele (C) rs3865444 risk variant of *CD33* was first identified in 2011 and is associated with elevated CD33 expression and reduced TREM2 expression in the brain as well as increased amyloid burden [66]. Functionally, studies show this variant to increase microglial activation and decrease Aβ phagocytosis. On the other hand, the minor allele (A) rs3865444 and rs12459419 variants yield a non-functional version of CD33 due to alternative splicing and loss of the sialic acid-binding domain, and have been described as a protective variants because they preserve the cell's ability to recognize, bind and clear Aβ [9,62,63,67–70].

CD33 (Siglec-3) is a member of the sialic acid-binding immunoglobulin-like lectins (Siglec) family of receptors, and recognizes sialic acid residues as its ligands, such as sialylated glycans found on pathogens [71]. *CD33* harbours an immunoreceptor tyrosine-based inhibitory motif (ITIM), which is the main route for inhibitory signal transduction in cells. ITIM-immunoreceptors in microglia are involved in the modulation of various cellular functions including phagocytosis, cytokine release and apoptosis. Upon ligand binding, the ITIM of CD33 is phosphorylated and acts as a docking site for phosphatases such as SHP1/2. Subsequently, this leads to downstream dephosphorylation of other cellular proteins such as PI3 K (that are conversely activated by ITAM signalling), leading to an inhibition of cellular activity and of functions such as phagocytosis [72–74]. Importantly, the aforementioned TREM2-ITAM signalling opposes CD33-ITIM signalling, and acts downstream of CD33 signalling [74].

Research indicates that AD brains have constitutively activated CD33 signalling, with microglial cells demonstrating an upregulation of CD33 expression that correlated with plaque burden [62]. Sialic-acid bearing glycoproteins and glycolipids are found to colocalize to and adorn amyloid plaques, thereby activating CD33 signalling. This in turn encourages ITIM-associated inhibitory signalling, resulting in the ‘masking’ of plaques against microglial recognition, thereby reducing phagocytosis and clearance. Additionally, CD33 signalling inhibits the release of inflammatory cytokines and proliferation by microglia, which are part of the microglial response to AD pathology [73,75]. Unsurprisingly, CD33 ablation in amyloid models of AD enhances phagocytosis and results in reduced Aβ plaque burden [62,74]. Targeting mouse CD33 using a miRNA in the APP/PS1 amyloid model has been seen to be effective at reducing plaque burden early on (two months), but not later in the disease (eight months) [76]. RNA-seq in the CD33^−/−^ 5xFAD mouse has revealed that genes related to phagocytosis, activation and cytokine signalling were upregulated, and that this was dependent on downstream TREM2 signalling [74]. Follow-up research is necessary to tease out the exact mechanism of interaction between CD33 and TREM2.

Independent groups have found that unlike its effects on amyloid, having higher CD33 brain expression is not associated with tau-pathology associated Braak score, and that carrying the major allele (C) rs3865444 allele seems to have no repercussions on tangle formation [77,78]. However, while little has been shown of the relationship between tau and CD33, NFTs have been shown to present sialic acid residues, suggesting a potential interaction [79].

### 2.3. APOE

APOE, while primarily secreted by astrocytes, is also produced by activated microglia surrounding amyloid plaques [33,80–83]. APOE polymorphic alleles (*ε*2, *ε*3, *ε*4) have been identified as critical genetic determinants of AD risk, with the APOE ε4 allele showing the strongest genetic risk for LOAD (and more common than FAD mutations), followed by ε3, while ε2 has displayed protective effects [84–86]. A single copy of APOE ε4 has been shown to increase the risk for developing AD 4-fold, while homozygous carriers show an approximate 12-fold increased risk for AD [84,85]. On the other hand, the rare APOE ε2 allele is protective [86]. It is still unclear whether the presence of the APOEε4 allele leads to a toxic gain of function or loss of protective function. As a secreted lipoprotein, apoE is involved in cholesterol metabolism. Moreover, it has also been identified as an amyloid-associated protein and found to be present abundantly in plaques, indicating a direct association between the two [33,80–83,87].

Studies in animal models of AD have suggested that apoE isoforms differentially impact Aβ deposition and clearance by microglia, as well as NFT formation [88,89]. As further proof of allelic contribution to AD risk, APOE alleles have been found to impact the efficacy of passive anti-Aβ immunization, suggesting that the different alleles could affect microglial phagocytic capabilities of Aβ [90]. Post-mortem analysis has indicated that both AD patients and healthy controls harbouring the APOE *ε*2/*ε*3 genotype have decreased amyloid deposition, whereas APOE*ε*4 carriers have more abundant amyloid [91]. Additionally, increased neuroinflammatory markers were found in APOE *ε*4 carriers and in corresponding mouse models. Further, apoE levels are lowest in APOE ε4 mouse models, and apoE is known to contribute to anti-inflammatory signalling [92]. Additionally, APOE *ε*4 primary microglia secrete 3–5 times less apoE and more TNFα than APOE *ε*2 microglia, suggesting that the presence of APOE *ε*4 drives microglia to a higher inflammatory state [93].

Differences in APOE allele function may be due to a variety of biochemical and physical changes induced by amino acid alterations. ApoE *ε*4 has reduced lipidation compared to ApoE *ε*3 and ApoE *ε*2, and also a lower affinity for apoE receptors, one of which is TREM2. Impaired binding of Aβ-apoE and TREM2 may potentially affect both microglial recognition of amyloid pathogens and subsequent clearance of plaques [94,95]. A recent *in vivo* study comparing the transcriptomic response of microglial cells exposed to either e3 or e4 lipoproteins along with Aβ, showed that the addition of *ε*3 lipoproteins to Aβ led to a more active transcriptional response and a higher upregulation of DAM genes in comparison to e4 lipoproteins. ε4-expressing microglia also showed reduced Aβ uptake, which was further aggravated by a TREM2 deficiency in these cells. Impaired binding of allelic variation in apoE thus contributes to differences in apoE-Aβ binding; Thus, the *ε*4 isoform is hypothesized to negatively impact TREM2-dependent Aβ binding in microglia, which may result in impaired microglial activation and a dampened phagocytic response. Lastly, the apoE *ε*4 isoform also induces a slower response by microglial processes toward apoE containing Aβ, suggesting apoE's contribution to microglial activation, motility or cytoskeleton reorganization [96]. Another study has shown that genetic risk variants TREM2 R47H and APOE *ε*4 act by reducing the responsiveness of microglia toward amyloid, which was associated with elevated pathology [42].

Additionally, while it is clear that *APOE* genotype contributes greatly to amyloid deposition, APOE's effect on tau remains contested. Human studies suggest that *APOE* *ε*4 carriers, in the presence of amyloid, have higher tau burdens in vulnerable AD brain regions, as opposed to non-carriers [97]. *In vivo* studies point to the ε4 allele as having the most deleterious effects in tau transgenic mice, opposed to *ε*2 and *ε*3. *APOE* *ε*4 has been shown to aggravate neurodegeneration and neuroinflammation in the PS19 tau transgenic mouse model, while APOE knockout is protective against neurodegeneration [98]. Further, this effect was shown to be specifically driven by microglia, as allelic differences contributed primarily to microglial activation and neurodegeneration, and complete ablation of microglia by PLX3397, an inhibitor of the colony-stimulating factor 1 receptor (CSF1R, necessary for microglial survival) in male APOE ε4 PS19 mice completely protected these mice from neurodegeneration [89,99]. More recently, Shi *et al.* [100] have demonstrated a novel apoE knockout model that overexpresses the apoE metabolic receptor *LDLR* (low-density lipoprotein receptor, responsible for mediating clearance of apoE lipoproteins) and that reduces brain apoE when crossed to PS19 mice, which results in decreased tau pathology and neurodegeneration. Nine month-old apoE KO transgenic mice showed reduced intracellular microglial apoE, as well as reduced microglial activation and CD68 staining. Suppression of microglial activation was found to be driven by enhanced microglial cell catabolism, as sequencing experiments highlighted enrichment of lysosomal enzymes and proteins involved in cellular degradation including *Lhmn*, *Ctss*, *Ctsk*, *Heb*, *Man2b1*, *Lamp1*, *Lamp2*, *Abca2*. Further, apoE deficiency was shown to reduce microglial mTOR activation, likely due to enhanced catabolic activity. Additionally, sequencing of cultured primary microglia from LDLR transgenic mice showed significant reduction in DAM and proinflammatory gene expression as well as MHC-related gene expression, and conversely, upregulation of ion channels and neurotransmitter receptors [100].

### 2.4. SPI1/PU.1

*SPI1*, implicated in LOAD through differential network analysis, is highly expressed in immune cells, specifically microglia and macrophages. Several variants at the SPI1 locus have been identified and studies linking risk variants to gene expression indicate that variants that confer higher SPI1 expression are linked to increased risk for AD. The minor allele (G) rs1057233 lies near the *SPI1* gene locus and is associated with lower expression of SPI1 in monocytes and macrophages and shows association with delayed AD onset [101]. It also influences the expression of other AD risk genes [14,102]. This SNP was previously identified in the context of systemic lupus erythematosus and was found to alter miRNA binding [103].

RNA sequencing data implicate *SPI1* in the AD immune response, as increased SPI1 is associated with the upregulation of AD-associated immune and interferon-response genes*. SPI1* encodes PU.1, a key transcription factor and master regulator of myeloid cell development and microglial gene expression and activation. In macrophages, PU.1 overexpression leads to increased GM-CSF and M-CSF expression (crucial factors for macrophage proliferation), as well as increased proliferation [104]. Transcriptomic analysis of reduced *PU.1* gene expression in primary glial cell cultures has highlighted PU.1's contribution to innate and adaptive immune responses, specifically in the involvement of antigen presentation and phagocytosis [105]. In the BV2 mouse microglial cell line, expression of microglial genes such as *Irf8*, *Runx1*, *Csf1r*, *Csf1*, *Il34*, *Aif1* (*Iba1*), *Cx3cr1*, *Trem2* and *Tyrobp*, some of which already cited in this article as risk factors for AD, were found to be regulated by *SPI1* [106].

Recent studies that have sought to characterize the effects of *PU.1* modulation *in vitro* using mouse BV2 cells have reported that increased PU.1 expression leads to cells becoming more resistant to cell death and more prone to converting to an inflammatory phenotype, which could be detrimental to neighbouring cells of the brain. On the other hand, *PU.1* knock-down had an opposing effect and made microglial cells more vulnerable to cell death, but also reduced inflammatory signalling. BV2 cells with reduced *PU.1* expression also had increased expression of lipid metabolism genes such as *ApoE*, and an overall repressed homeostatic gene expression profile, which aligned to the DAM signature described earlier in this review [107]. In line with previous reports, *PU.1*-overexpressing cells showed increased uptake of a variety of substrates such as zymosan, myelin and apoptotic cells [9,101,105,107]. Additionally, silencing *PU.1* in primary human microglia results in changes in gene expression, particularly in a network of AD-associated genes involved in immune functions, such as phagocytosis and antigen presentation [105]. Overall, these studies validate *PU.1* as a positive regulator of phagocytic uptake and suggest that PU.1 overexpression primes microglial cells for an exaggerated inflammatory immune response. Further, while the aforementioned research provides promising *in vitro* results, further *in vivo* and *in situ* data are necessary to fully characterize the role of *PU.1* in a more relevant disease model. The current hypothesis is that reduced expression of *PU.1* may be beneficial due to an increased turnover and replenishment of microglial cells following apoptotic cell death in response to neuropathology, as well as turning on a more active and protective state. Further work is necessary to define the role of *SPI1/PU.1* in tauopathy.

### 2.5. MS4A

The membrane-spanning 4-domain subfamily A (*MS4A*) gene cluster harbours 18 genes, of which *MS4A4A*, *MS4A4E*, *MS4A6A* and *MS4A6E* have been implicated in AD [9,69]. The main SNPs that have been identified as having an association with AD are rs670139 in *MS4A4E*, rs4938933 and rs1562990 within the region between *MS4A4E* and *MS4A4A*, and rs610932 in *MS4A6A* [68,108,109]; increased expression of *MS4A6A* is associated with higher amyloid plaque and neurofibrillary tau tangle burden [77]. Further, binding motifs within *MS4A4A* and *MS4A6A* have been identified for transcription factor PU.1, and PU.1/SPI1 correlate closely with changes in *MS4A4A* and *MS4A6A* [77]*.* MS4A transmembrane proteins are found to be expressed in microglia and macrophages, as well as in peripheral immune cells. While their function in the brain is still poorly understood, they have been cited for their roles in calcium homeostasis, endocytosis and trafficking and cell signalling [110,111].

MS4A proteins are known to be involved in calcium signalling. MS4A1 is part of the Ca^2+^-permeable cation channel, and MS4A2 regulates mitochondrial Ca^2+^ uptake and increases downstream calcium signalling [112,113]. Given their conserved protein structure, other members of the MS4A family may share similar functions. Alterations in calcium signalling during the early stages of AD have also been seen in human subjects and experimental mouse models [114,115].

The *MS4A* gene cluster has also been reported to be involved in brain immune system function. Overexpression of the *MS4A* gene family increases T cell activation, and also regulates apoptosis and survival of activated T cells [116,117]. Activated T cells have been found in the healthy brain as well as under neuroinflammatory conditions, and T cell activation can influence the trafficking of additional T cells across the blood--brain barrier (BBB). This in turn has been shown to increase microglial activation and production of inflammatory cytokines during AD progression [118,119].

More recently, genome-wide analysis for genetic modifiers of CSF sTREM2 has identified two SNPS in the *MS4A4A* gene that modify CSF sTREM2 concentrations. Rs1582763 was found to be associated with elevated CSF sTREM2 and reduced AD risk. It has previously been associated with delayed onset of AD [9,101]. On the other hand, rs6591561 was found to be associated with reduced CSF sTREM2 and increased AD risk and accelerated onset. Functional studies in human macrophage cultures provide support for a modulatory relationship between *MS4A4A* and sTREM2, with *MS4A4A* overexpression resulting in elevated sTREM2 and *vice versa*. MS4A4A was also found to colocalize with TREM2 in the cytoplasm of human macrophages, further validating an interaction between the two proteins [56]. Overall, these findings suggest that MS4A4A may promote TREM2 processing and subsequent microglial signalling and thus play a role in LOAD-pathogenesis. In addition, MS4A2 is known to contain an ITAM motif in its protein sequence, which may potentially lead to downstream activation signalling in microglia [120,121].

### 2.6. ABCA7

GWAS have identified the ATP-binding cassette transporter A7 (*ABCA7*) as a risk gene for LOAD. Both common and rare risk variants in *ABCA7*, including intronic, VNTR and PTC mutations have been found to be enriched in AD patients, with loss-of-function variants increasing disease risk [121,122]. Both genetic and epigenetic ABCA7 markers show significant correlation with AD endophenotypes including amyloid deposition, brain atrophy and cognitive decline [68,122,123].

ABCA7 is part of an ABC transporter superfamily and is involved in lipid metabolism, specifically in the transfer of phospholipids to apolipoproteins (i.e. APOE and APOJ/CLU) and in the transport of lipids across membranes [106,124–126]. ABCA7 knockout mice display altered brain phospholipid profiles. Moreover, genome-wide analysis of genetically modified ABCA7 mouse models has identified an enrichment of cellular membrane homeostasis pathways [122]. Altered lipid metabolism is likely to affect endolysosomal pathways through functions such as vesicle trafficking and is likely to affect phagocytic and degradative capabilities.

The highest expression of ABCA7 in the brain has been found in microglia. Recent research has brought to light ABCA7's involvement in microglial phagocytosis [127], since ABC transporters show high homology to *ced-7*, the cell corpse engulfment gene in *Caenorhabditis elegans* known to phagocytose apoptotic cells [128]. Studies show that ABCA7^−/−^ peritoneal macrophages and immune cells in ABCA7 knockout mice have diminished phagocytic capabilities [129]. ABCA7 deletion in AD mouse models has shown increased amyloid deposition and decreased phagocytic uptake of oligomeric Aβ_1–40_ and Aβ_1–42_ in both macrophages and microglia, without changing microglial activation status [122,130]. Further, increased levels of ABCA7 promote microglial phagocytosis and clearance of Aβ, presumably through the C1q complement pathway [106,107,122,124,130]. Interestingly, the involvement of ABCA7 in microglial phagocytosis is thought to affect Aβ aggregates rather than soluble Aβ, as evidenced by microdialysis studies [122].

**Figure 2.**
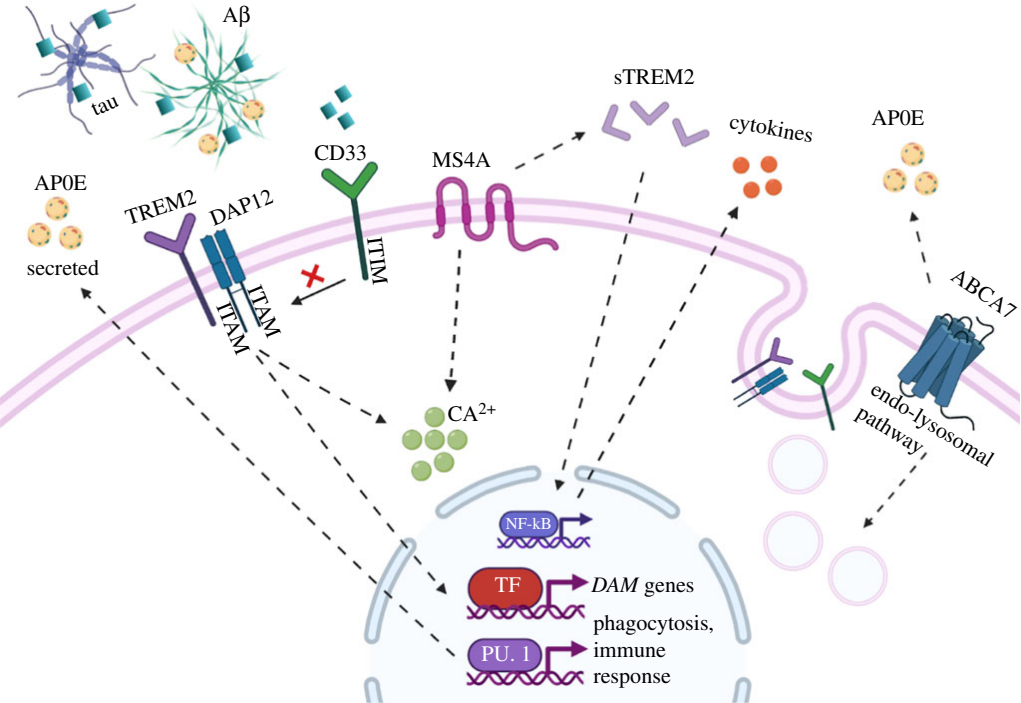
Putative functions of LOAD-associated genes in microglial cells. Microglial response to pathogenic targets such as Aβ and tau begins with initial recognition via receptors such as TREM2. TREM2 signals through DAP12 to affect intracellular calcium signalling and *DAM* gene expression, which leads to functional outputs such as inflammatory signalling and phagocytic uptake of targets. CD33 detects sialylated targets (blue squares) and is an inhibitor of phagocytosis and opposes TREM2 signalling. sTREM2 is cleaved from full-length TREM2 and is also involved in NF-kB signalling and inflammatory response. MS4A is a transmembrane protein that is known to influence calcium signalling and modulate TREM2 processing into sTREM2. ABCA7 is a transporter protein and there is evidence that it affects the endolysosomal function and metabolism of lipids, which can affect APOE. APOE is involved in cholesterol metabolism and is found to associate with Aβ plaques, affecting their interaction with TREM2. (Created with BioRender.com.)

Aikawa *et al*.'s research further implicates ABCA7 in the microglial immune response to AD pathogenesis. ABCA7 haplodeficiency in mice was shown to be associated with increased Aβ and CD14 accumulation in microglial cells within enlarged lysosomes. This dysregulation of CD14 trafficking potentially leads to a reduced activation of the NF-kB pathway, without affecting the expression of proinflammatory cytokines and DAM markers [131] (figure 2).

### 2.7. Additional microglial-related LOAD genes

Additionally, several other genes identified by GWAS have been reviewed for their immune-related roles in AD, including *CR1*, *ApoJ/Clu* and *PLCG2*, yet more studies are still necessary to identify specific functional contributions. Briefly, complement receptor 1 (CR1) expressed in glial cell populations, has been identified through GWAS as a risk factor for AD; complement factors are highly reviewed for their immune-related contribution to AD. Moreover, microglial expression of complement proteins and receptors has been shown to play a critical role in dystrophic neurite and pathogen recognition and clearance [132]. CR1 acts as the key receptor for complement protein C3B, and studies show Aβ42 binding of C3b, ultimately bridging Aβ to CR1 and phagocytes. Inhibition of CR1 reduces microglial phagocytosis of Aβ and studies link AD-related mutations in CR1 to decreased Aβ clearance in CSF [133]. Apolipoprotein J (*apoJ*, also known as clusterin/*Clu*) is also a risk variant in AD [108]. Studies show upregulation of apoJ to be associated with amyloid plaques, NFT-positive dystrophic neurites and surrounding activated microglia in the AD brain [43]. Apolipoproteins have been associated with Aβ fibrillization and opsonization-related clearance; apoJ has been reported to bind soluble Aβ and Aβ aggregates [134,135]. Researchers have found that microglia may engulf Aβ more efficiently in the presence of lipoproteins including LDL and apoJ, and that the uptake of lipoprotein–Aβ complexes by microglia is TREM2-dependent. ApoJ is a reported ligand of TREM2, and TREM2^−/−^ microglia show reduced internalization of apoJ [43,136]. Moreover, *in vitro* and *in vivo* studies have shown that exogenous apoJ activates microglia [137]. Additionally, PLCG2, a member of the phospholipase Cy family, is highly expressed in microglial cells. Identification of a PLCG2 rare variant, P522R, is associated with decreased risk of AD; this polymorphism results in an increase in PLCy2 enzyme activity [138]. Expression of PLCG2 is increased in microglia surrounding amyloid plaques [139]. Further, PLCG2 is also known to colocalize with TREM2, and is assumed to be involved in TREM2 downstream signalling [139,140].

Further, GWAS has identified other genes such as SORL1 for potential roles in microglial phagocytosis, while other genes such as GRN and PICALM are implicated in endolysosomal regulation and pathogen degradation. Functional studies are still necessary to piece apart the exact roles of these genes in microglial-related pathways [10,13]. Moving forward, in order to understand the contribution of novel immune gene variants in LOAD, more relevant models of disease must be assessed [141]. Currently, the majority of AD mouse models focus on the expression of rare familial amyloid-related mutations in APP and Presenilin, or of tau-related gene overexpression [142]. However, newly identified rare and common risk variants in LOAD require novel mouse models for further investigation [16,83,143].

## 3. Conclusion

As outlined in this review, genetic studies (i.e. GWAS, expression network analyses) of AD pathogenesis have underscored the significance of microglia in LOAD development and progression. Research has shown that identified gene variants in microglia have roles in a variety of microglial functions, including but not limited to pathogen identification, phagocytosis, phagolysosomal digestion and immune signalling. Moreover, work in human and AD mouse models has shown that these genes (i.e. *Trem2*) can play conflicting roles, protective or detrimental, depending on the stage of disease development and progression, making it critical to understand the specific time-dependent roles in effects of risk variants in AD [144]. Functional genomics as well as the identification of coding gene variants thus provide a framework of support for the hypothesis that microglia play a causative role in AD development, rather than a purely responsive reaction triggered by AD pathology.

Furthermore, the majority of research on AD therapeutics has been geared towards targeting AD hallmarks (i.e. amyloid and tau); however, emerging data have unveiled novel pathways in immune cell function, ultimately helping shed light on alternative routes for therapeutic intervention. Preliminary research has helped to unpack some of the functions associated with AD risk genes. However, continued exploration is necessary to better comprehend the full functional spectrum of disease variants in microglia, for the purpose of identifying novel and effective targets for AD therapy.

## Data accessibility

This article has no additional information.
